# Effects of tofacitinib on the clinical features of periodontitis in patients with rheumatoid arthritis: two case reports

**DOI:** 10.1186/s41927-019-0062-y

**Published:** 2019-04-19

**Authors:** Tetsuo Kobayashi, Satoshi Ito, Akira Murasawa, Hajime Ishikawa, Hiromasa Yoshie

**Affiliations:** 1 General Dentistry and Clinical Education Unit, Niigata University Medical and Dental Hospital, 2-5274 Gakkocho-dori, Chuo-ku, Niigata, 951-8514 Japan; 2 Division of Periodontology, Department of Oral Biological Science, Niigata University Graduate School of Medical and Dental Sciences, 2-5274 Gakkocho-dori, Chuo-ku, Niigata, 951-8514 Japan; 3 Department of Rheumatology, Niigata Rheumatic Center, 1-2-8 Honcho, Shibata, 957-0054 Japan

**Keywords:** Rheumatoid arthritis, Periodontitis, Tofacitinib, Proinflammatory cytokines, Case report

## Abstract

**Background:**

The pathobiology of rheumatoid arthritis (RA) is similar to that of periodontitis in that proinflammatory cytokines play an important pathologic role. There is evidence to suggest that inhibitors of tumor necrosis factor (TNF) and interleukin-6 (IL-6) receptor for the treatment of RA ameliorated periodontal inflammation. However, no study has evaluated the effect of tofacitinib, an oral Janus kinase inhibitor for the treatment of RA, on periodontitis.

**Case presentation:**

The present report cases are 51- and 43-year-old non-smoking women with RA who demonstrated localized moderate chronic periodontitis. Both cases showed improvement in the periodontal inflammatory condition after 3 months of tofacitinib therapy, although the teeth count and supragingival bacterial plaque level were relatively unchanged. Improvements were also observed in the serum levels of IL-6 in both cases as well as in the serum levels of TNF-α and anti-cyclic citrullinated peptide immunoglobulin G in one case and of rheumatoid factor and matrix metalloproteinase-3 in the other case. Patients who received tofacitinib exhibited an inconsistent clinical response, likely due to the low disease activity of RA at the start of the administration.

**Conclusions:**

These are the first reported cases in which tofacitinib may have a beneficial effect on periodontitis. However, more research is required to understand the relationship between periodontitis and tofacitinib therapy.

## Background

Rheumatoid arthritis (RA) is a chronic inflammatory joint disease that can cause damage to the cartilage and bone as well as disability [1]. Evidence suggests that RA has an epidemiological, serological, and clinical interrelationship with periodontitis, a chronic inflammatory disease that is characterized by the destruction of the tooth-supporting tissues and is a major cause of tooth loss in adults, through common pathogenic mechanisms [1–4]. One of these mechanisms includes the constitutive overproduction of proinflammatory cytokines, including tumor necrosis factor-alpha (TNF-α) and interleukin-6 (IL-6), both of which are involved in the pathogenesis of RA and periodontitis [5–7].

Studies have shown that inhibitors of TNF-α and IL-6 receptor not only reduce the signs and symptoms of RA but also ameliorate the periodontal inflammatory conditions in patients with RA [7, 8]. Other studies also suggest the efficacy of targeting intracellular pathways in inhibiting the effects of multiple cytokines [9, 10]. Tofacitinib, an oral small-molecule inhibitor for Janus kinase (JAK) that integrates signals from many cytokines, has been shown to be effective in the treatment of RA [11, 12]. These observations have led to the hypothesis that tofacitinib may also be effective in reducing periodontal inflammation in patients with RA. However, no study has yet documented the effect of tofacitinib on periodontitis.

The aim of the present study was therefore to report the changes in the periodontal inflammatory condition before (baseline) and after 3 months (reassessment) of tofacitinib therapy in two patients with RA.

## Case presentation

*Case 1:* The patient was a 51-year-old non-smoking woman with a 68-month history of RA. Before tofacitinib was administered, she had been treated with prednisolone (PSL, 10 mg/day) and bucillamine (BUC, 200 mg/day) for 13 months and was then switched to receive the recombinant humanized anti-human IL-6 receptor monoclonal antibody tocilizumab (TCZ, 8 mg/kg, every 4 weeks) intravenously. Under this treatment, her disease activity score in 28 joints using C-reactive protein (DAS28-CRP) was well controlled as follows: from 3.8 (the baseline) to 2.5 (after 4 months of treatment). However, TCZ was discontinued after 4 months due to signs of pneumonia in the right lung. She was then switched to the fully humanized anti-TNF-α monoclonal antibody adalimumab (ADA, 40 mg/2 weeks) subcutaneously, which resulted in a well-controlled DAS28-CRP for 34 months as follows: from 3.9 (the baseline) to 1.4 (after 34 months of treatment). She was then transferred to a local rheumatology clinic and showed a similar RA condition for 8 months with 5 mg/day of PSL and 12 mg/week of methotrexate (MTX). However, she returned to visit to the Niigata Rheumatic Center with joint pain and swelling. Her CRP levels were gradually raised and at the last visit of that clinic, it was 3.45 mg/dL. The clinical and laboratory assessments at our rheumatic center revealed DAS28-CRP 4.32 and global visual analogue scale (gVAS) 28, possibly due to the secondary failure of the response to ADA treatment. Two weeks later, we evaluated her periodontal condition and started the administration of tofacitinib (10 mg/day) according to the European League Against Rheumatism recommendations for the management of RA [13]. For some reason, her CRP level was decreased to 0.1 mg/dL, but her gVAS was worsened to 51 (Table 1). The patient had no complications, such as hypertension or systemic viral infections, at baseline.

**Table 1:** Patient rheumatologic and serum data at baseline and reassessment

Parameter	Case 1		Case 2	
	Baseline	Reassessment	Baseline	Reassessment
Rheumatologic				
SDAI	12.3	4.5	16.0	4.0
DAS28-CRP	2.3	2.9	2.9	2.1
TJC	2	3	3	1
SJC	0	0	4	1
gVAS (mm)	51	7	45	10
Serum				
RF (IU/mL)	5.0	7.0	237.0	174.0
anti-CCP IgG (U/mL)	37.8	25.7	247.0	301.0
CRP (mg/dL)	0.1	0.1	0.01	0.01
MMP-3 (ng/mL)	67.7	70.0	49.9	44.3
IL-6 (pg/mL)	2.2	1.5	1.9	0.9
TNF-α (pg/mL)	1.2	0.0	1.0	0.9

The rheumatologic assessments showed a decrease in the simplified disease activity index (SDAI) and gVAS at reassessment after starting tofacitinib therapy (Table 1). In addition, the laboratory analyses of blood samples showed that the serum levels of anti-cyclic citrullinated peptide (CCP) immunoglobulin G (IgG), TNF-α, and IL-6 were decreased at reassessment compared to the values at baseline (Table 1).

**Fig. 1:**
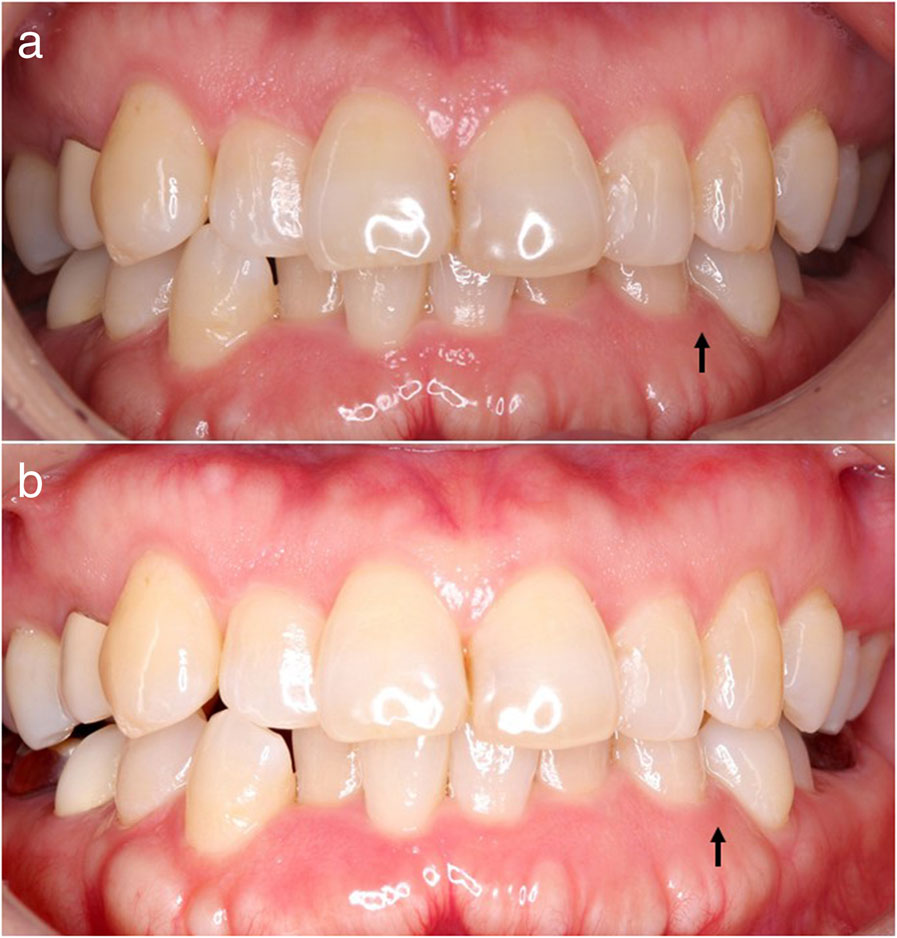
Photographs of case 1, demonstrating localized moderate chronic periodontitis **a** before (baseline) and **b** after 3 months (reassessment) of tofacitinib therapy. Improvements were observed in the gingival index (GI), probing depth (PD), and clinical attachment level (CAL) at the periodontitis-affected site with the black arrow at **b** (reassessment) compared to those at **a** (baseline), although the supragingival bacterial plaque level as defined by the plaque control record (PCR) was relatively unchanged (baseline to reassessment: 2 to 0 for GI; 4 mm to 2 mm for both PD and CAL)

Furthermore, the periodontal assessments indicated that the patient had localized moderate chronic periodontitis at baseline according to the criteria of the Centers for Disease Control and Prevention (CDC)/American Academy of Periodontology (AAP) [14] (Fig. 1a). Tofacitinib therapy reduced periodontal inflammation as indicated by the mean values of the gingival index (GI), probing depth (PD), and clinical attachment level (CAL), as well as the percentage of sites with bleeding on probing (BOP) and of those with PD and CAL of ≥4 mm at reassessment, although the teeth count and supragingival bacterial plaque level as defined by the plaque control record (PCR) were relatively unchanged after tofacitinib therapy (Fig. 1b and Table 2).

**Table 2:** Patient periodontal data at baseline and reassessment

Parameter	Case 1		Case 2	
	Baseline	Reassessment	Baseline	Reassessment
Teeth count	26	26	20	20
PCR (%)	11.5	12.5	57.5	55.0
Mean GI	0.5	0.2	0.8	0.3
BOP (%)	3.9	0.6	8.3	0.0
Mean PD (mm)	2.8	2.0	3.0	2.2
PD ≥ 4 mm (%)	10.3	0.0	25.0	1.7
Mean CAL (mm)	2.8	2.0	3.1	2.3
CAL ≥ 4 mm (%)	10.3	0.0	25.0	1.7

*Case 2:* The patient was a 43-year-old non-smoking woman with a 39-month history of RA. Before tofacitinib was administered, she had been treated with MTX (4 mg/week) and BUC (100 mg/day), and the DAS28-CRP was well controlled for 29 months as follows: from 2.0 (the baseline) to 1.2 (after 29 months of treatment). However, because of the lack of a response to the treatment with MTX and BUC, the further administration of tofacitinib (10 mg/day) was started. The patient had no complications, such as diabetes mellitus, hypertension, or systemic viral infections, at baseline.

The rheumatologic assessments showed a decrease in the SDAI, DAS28-CRP, tender joint count (TJC), swollen joint count (SJC), and gVAS at reassessment after starting tofacitinib therapy (Table 1). The laboratory analyses of blood samples showed that the serum levels of rheumatoid factor (RF), matrix metalloproteinase-3 (MMP-3), and IL-6 were decreased at reassessment compared to the values at baseline (Table 1).

Furthermore, the periodontal assessments indicated that the patient had localized moderate chronic periodontitis at baseline according to the criteria of the CDC/AAP [14]. Tofacitinib therapy reduced periodontal inflammation as indicated by the mean values of the GI, PD, and CAL, as well as the percentage of sites with BOP and of those with PD and CAL of ≥4 mm at reassessment, although the teeth count and supragingival bacterial plaque level as defined by the PCR were relatively unchanged after tofacitinib therapy (Table 2).

## Discussion

These are the first reported cases in which tofacitinib may have a beneficial effect on periodontitis. Tofacitinib therapy reduced the SDAI, gVAS, and the serum levels of IL-6 in both cases. In particular, tofacitinib has been shown to be efficacious in improving pain as indicated by patient’s assessment [15]. These observations are consistent with the results of other studies that indicated the efficacy of tofacitinib in relieving the rheumatologic condition [11, 12] and showed that tofacitinib was able to suppress IL-6 signaling directly [10]. Improvements were also observed in the serum levels of TNF-α and anti-CCP IgG in one case and in those of RF and MMP-3 in the other case. Patients who received tofacitinib exhibited an inconsistent clinical response, likely due to the low disease activity of RA at the start of the administration.

Notably, both cases also exhibited a decrease in periodontal inflammation, although their teeth count, bacterial plaque level, and RA medication were relatively unchanged after tofacitinib therapy. Rheumatologists and periodontists were blinded regarding the rheumatologic and periodontal conditions as well as the administration of tofacitinib. Corticosteroid and non-steroidal anti-inflammatory drugs have little beneficial effect on periodontitis [16], and the clinical effects of MTX on periodontitis have not been studied. Therefore, the reduction in periodontal inflammation is likely due to the administration of tofacitinib rather than to any changes in periodontitis-related risk factors, such as bacterial plaque and smoking habit. This efficacy of tofacitinib might be due to the suppression of IL-6 signaling [10], which has been shown to be related to the reduction in periodontal inflammation [7, 8, 17, 18]. However, we were unable to evaluate these JAK-mediated signaling of cytokines in periodontal tissue due to the ethical limitations. In addition, improvements in the clinical periodontal conditions resulted in changes in both case definitions from baseline to reassessment (from moderate to no periodontitis for case 1 and from moderate to mild periodontitis for case 2) according to the criteria of the CDC/AAP [14].

## Conclusions

The present two cases demonstrated for the first time that tofacitinib may have a beneficial effect on periodontitis. However, more research is required to understand the relationship between periodontitis and tofacitinib therapy.

## Abbreviations

AAP: American Academy of Periodontology
ADA: Adalimumab
BOP: Bleeding on probing
BUC: Bucillamine
CAL: Clinical attachment level
CCP: Cyclic citrullinated peptide
CDC: Centers for Disease Control and Prevention
DAS28-CRP: Disease activity score in 28 joints using C-reactive protein
GI: Gingival index
gVAS: global visual analogue scale
IgG: Immunoglobulin G
IL-6: Interleukin-6
JAK: Janus kinase
MMP-3: Matrix metalloproteinase-3
MTX: Methotrexate
PCR: Plaque control record
PD: Probing depth
PSL: Prednisolone
RA: Rheumatoid arthritis
RF: Rheumatoid factor
SDAI: Simplified disease activity index
SJC: Swollen joint count
TCZ: Tocilizumab
TJC: Tender joint count
TNF: Tumor necrosis factor
